# Association of the neutrophil percentage-to-albumin ratio after endovascular treatment and 3-month clinical outcomes

**DOI:** 10.3389/fneur.2026.1768949

**Published:** 2026-02-12

**Authors:** Chang Cui, Liang Liu, Hui-Sheng Chen

**Affiliations:** Department of Neurology, General Hospital of Northern Theater Command, Shenyang, China

**Keywords:** acute ischemic stroke, clinical outcome, endovascular treatment, large vessel occlusion, neutrophil percentage-to-albumin ratio

## Abstract

**Background and aim:**

Neutrophil percentage-to-albumin ratio (NPAR) is associated with clinical outcomes in malignancy, cardiovascular disease, and stroke. This study aimed to evaluate whether NPAR levels are associated with clinical outcomes in patients with acute ischemic stroke (AIS) due to large vessel occlusion (LVO) who underwent endovascular treatment (EVT).

**Methods:**

From a prospective cohort, we consecutively enrolled patients with anterior circulation LVO-AIS who underwent EVT and had available admission and 48-h post-EVT NPAR data. Three-month clinical outcome was assessed using the modified Rankin Scale (mRS). Poor functional outcome was defined as functional dependence or death (mRS 3–6). Multivariable logistic regression analyses were performed to explore the relationship between NPAR levels and clinical outcomes.

**Results:**

A total of 121 eligible patients were included in the final analysis. Multivariable logistic regression models showed that a higher 48-h NPAR level (Model 1: adjusted OR = 15.09, 95% CI 3.72–61.22, *p* < 0.001) and an increase in NPAR from admission to 48 h (Model 2: adjusted OR = 7.69, 95% CI 2.06–28.70, *p* = 0.002) were independently associated with poorer functional outcome. The optimal cutoff value of 48-h NPAR level for predicting poor functional outcome was 2.312, with a sensitivity of 66% and a specificity of 79%.

**Conclusion:**

Higher 48-h follow-up NPAR levels and increases in NPAR from admission to 48 h were independent predictors of poor 3-month functional outcomes in patients with large vessel occlusion treated with endovascular therapy.

**Clinical trial registration:**

https://www.clinicaltrials.gov, identifier (NCT05092139).

## Introduction

Endovascular treatment (EVT) has become the standard of care for acute ischemic stroke (AIS) due to large vessel occlusion (LVO); however, significant challenges remain (1). A key challenge is that, even with ongoing refinements in EVT techniques yielding successful reperfusion rates as high as 84–88%, a substantial proportion of patients still fail to achieve functional independence at 90 days (2, 3). Accurately predicting which patients will experience functional dependence at 90 days remains an important clinical objective.

Post-stroke inflammation plays a critical role in secondary brain injury, contributing to blood–brain barrier disruption, cerebral edema, and reperfusion injury (4). Neutrophils are among the first immune cells recruited to the ischemic brain and can exacerbate tissue damage through degranulation, oxidative stress, and the formation of neutrophil extracellular traps (NETs) (5–7). In contrast, serum albumin exerts neuroprotective effects by maintaining plasma osmotic pressure, scavenging free radicals, and mitigating inflammatory cascades (8). An integrated index derived from neutrophil and albumin levels reflects both inflammatory activation and nutritional status, and may serve as a sensitive prognostic indicator in patients with AIS. Compared with absolute neutrophil count, neutrophil percentage better reflects the relative dominance of neutrophils within the circulating leukocyte pool and may be less influenced by acute hemodilution. Notably, the neutrophil percentage-to-albumin ratio (NPAR) has recently shown promise as a potential predictor of functional outcomes in both ischemic and hemorrhagic stroke (9–11).

Nevertheless, the prognostic value of perioperative NPAR levels in LVO-AIS patients treated with EVT remains unclear. Moreover, temporal profiling of NPAR following EVT may provide additional insights into its relationship with clinical outcomes in this population, which has not yet been thoroughly investigated (12). Therefore, this study aimed to investigate whether both admission (pre-EVT) and follow-up (within 48 h after EVT) NPAR levels are associated with 3-month clinical outcome in LVO-AIS patients.

## Methods

### Study design and population

We used data from the Endovascular Treatment for Acute Ischemic Stroke in China (DETECT2-China) registry, a multicenter, prospective, observational study evaluating patients with AIS treated with EVT at 21 comprehensive stroke centers in China. The design and methodology of the registry have been described in detail in the published protocol (13). Written informed consent was obtained from all patients or their legal representatives. The DETECT2-China trial is registered on ClinicalTrials.gov (NCT05092139). This observational study was conducted in accordance with the STROBE guidelines (14).

For the present study, we consecutively enrolled patients with AIS who underwent EVT at a large tertiary comprehensive hospital between January 2022 and October 2024, using data from the DETECT2-China registry. The inclusion criteria were as follows: (1) age ≥18 years, (2) clinically confirmed acute anterior circulation ischemic stroke with LVO, (3) treatment with EVT, and (4) availability of serum neutrophil percentage and albumin data at admission and within 48 h after EVT. The exclusion criteria were as follows: (1) clinically confirmed acute posterior circulation ischemic stroke; (2) history of terminal cancer, hematological disease, recent major trauma or surgery, or severe hepatic or renal disease as determined by clinical history or laboratory data; (3) use of immunosuppressive agents; (4) active infection within 2 weeks prior to admission; and (5) absence of 90-day follow-up data. The study was approved by the institutional review board of the General Hospital of Northern Theater Command (IRB: 2021–077) and was conducted in accordance with the Declaration of Helsinki.

### Data collection

Demographic and clinical data, including imaging and laboratory results, vascular risk factors, and past medical history, were collected. Venous blood samples were obtained on admission and within 48 h after EVT. NPAR was calculated as: neutrophil percentage (%) × 100/serum albumin (g/dL). For patients with two or more blood samples collected within 48 h, the neutrophil percentage was defined as the maximum value, while the albumin level was defined as the minimum value observed during this period. The 48-h NPAR was selected based on the temporal trajectory and peak timing of post-stroke inflammatory changes reported in previous studies (15, 16).

### Study outcome

Three-month clinical outcomes were assessed using the modified Rankin Scale (mRS) by trained staff. Poor functional outcome was defined as functional dependence or death (mRS 3–6), while good functional outcomes were defined as an mRS score of 0–2.

### Statistical analysis

Continuous variables were compared using the independent-samples *t*-test or the Mann–Whitney *U*-test, as appropriate, based on the Kolmogorov–Smirnov test for normality. Categorical variables were summarized as frequencies and percentages, and group differences were evaluated using the chi-square test. Spearman’s rank correlation coefficient was used for univariate correlation analysis. Univariable logistic regression was performed to identify risk factors associated with poor functional outcome at 90 days. Variables with a *p*-value of <0.05 were considered potential predictors. Multicollinearity among these variables was assessed using the variance inflation factor (VIF), with a VIF of <3 indicating no significant collinearity. Qualified variables were subsequently entered into a multivariable logistic regression model to determine independent factors associated with poor functional outcome at 90 days after endovascular thrombectomy (EVT). Receiver operating characteristic (ROC) curves were used to test the overall discriminative ability of the NPAR for poor functional outcome and to establish optimal cutoff points at which the sum of the specificity and sensitivity was highest. The *p*-values for ROC analyses were derived from pairwise comparisons of AUCs using the DeLong test, with admission NPAR as the reference. For handling missing data, multiple imputation by chained equations (MICEs) was used to generate 10 imputed datasets. The imputation model included all covariates used in the multivariable logistic analyses. Variables without missing values and the outcome variable were included only as predictors in the imputation process and were not imputed. Estimates derived from the imputed datasets were pooled using Rubin’s rules, accounting for both within- and between-imputation variability. All statistical analyses were performed with SPSS software, version 29 (IBM), and R software, version 4.3.3 (R Foundation). All *p*-values were two-sided, and a *p*-value of <0.05 was considered statistically significant.

## Results

### Baseline characteristics

A total of 586 patients were screened, of whom 121 eligible patients were included after the exclusion of 465 patients (Supplementary Figure S1). As shown in Table 1, the median age of the included patients was 67 years (IQR, 58–71), and 75.2% (91/121) were male. Vascular risk factors were common: 55.4% (67/121) had hypertension, 24.0% (29/121) had diabetes, and 33.1% (40/121) had a history of prior stroke/TIA. The median baseline NIHSS score was 14 (IQR, 11–17), and the median ASPECTS was 8 (IQR, 7–9). The majority of patients achieved successful reperfusion [94.2% (114/121)]. The median onset-to-puncture time was 446 min (IQR, 290–833). The predominant first-line EVT approach was a combined stent retriever plus aspiration technique (73.6%).

**Table 1:** Baseline characteristics of the study population.

Variable	Total (*n* = 121)	Poor outcome (*n* = 39)	Good outcome (*n* = 82)	*p*-value
Age	67.0 (58.0–71.0)	70.0 (61.5–74.0)	64.0 (57.2–69.8)	0.019
Male	91 (75.2%)	33 (84.6%)	58 (70.7%)	0.098
Vascular risk factors				
Current smoker	38 (31.4%)	11 (28.2%)	27 (32.9%)	0.601
Current drinker	15 (12.4%)	5 (12.8%)	10 (12.2%)	1.000
Hypertension	67 (55.4%)	23 (59.0%)	44 (53.7%)	0.582
Diabetes	29 (24.0%)	11 (28.2%)	18 (22.0%)	0.451
Atrial fibrillation	21 (17.4%)	9 (23.1%)	12 (14.6%)	0.252
Prior stroke or TIA	40 (33.1%)	15 (38.5%)	25 (30.5%)	0.384
Pre-mRS 1 or 2	10 (8.3%)	4 (10.3%)	6 (7.3%)	0.845
ASPECTS	8.0 (6.8–9.0)	7.0 (5.0–8.5)	8.0 (7.0–9.0)	0.021
Baseline SBP	147.7 (24.0)	149.6 (28.6)	146.8 (21.6)	0.587
Baseline NIHSS	14.0 (11.0–17.0)	17.0 (12.0–18.0)	12.5 (11.0–16.8)	<0.001
Admission laboratory data				
Blood glucose (mg/dL)	133 (112–162)	146 (119–174)	131 (108–152)	0.048
Neutrophil percentage	76 (67–84)	78.0 (68–85)	76 (67–83)	0.378
Albumin (g/L)	41.5 (3.9)	40.8 (4.0)	41.8 (3.9)	0.218
Platelet	209 (171–267)	205 (152–259)	210 (178–276)	0.191
Hemoglobin (g/L)	148 (136–159)	147 (135–160)	148 (137–157)	0.993
Fibrinogen (g/L)	2.8 (2.4–3.5)	3.0 (2.4–3.7)	2.8 (2.4–3.4)	0.288
ALT (U/L)	21.2 (16.2–30.8)	24.1 (17.7–32.6)	20.3 (15.7–29.1)	0.159
AST (U/L)	23.0 (19.5–28.0)	26.1 (21.9–35.2)	21.9 (19.2–25.8)	0.007
Total protein (g/L)	69.7 (5.7)	69.7 (5.3)	69.7 (5.9)	0.954
Globulin (g/L)	27.8 (25.0–30.7)	28.5 (25.1–32.3)	27.7 (25.2–30.4)	0.346
Creatinine (μmol/L)	67.7 (58.4–78.8)	72.7 (60.5–84.1)	65.2 (56.2–74.3)	0.063
48-h laboratory data				
Neutrophil percentage	80.0 (76.0–87.0)	86.0 (80.0–89.0)	78.5 (75.0–85.0)	<0.001
Albumin (g/L)	36.7 (4.5)	34.4 (4.8)	37.7 (4.0)	<0.001
NPAR levels				
Admission	1.8 (0.3)	1.9 (0.4)	1.8 (0.3)	0.250
At 48 h	2.2 (2.0–2.4)	2.4 (2.2–2.8)	2.1 (1.9–2.3)	<0.001
Change from admission	0.4 (0.2–0.6)	0.6 (0.3–0.9)	0.3 (0.1–0.5)	<0.001
Intravenous thrombolysis	24 (19.8%)	5 (12.8%)	19 (23.2%)	0.182
Workflow times (min)				
OTT	216 (156–257)	234 (223–236)	183 (154–262)	0.754
OPT	446 (290–833)	405 (311–793)	485 (286–843)	0.440
ORT	503 (330–901)	482 (335–883)	535 (330–929)	0.614
PRT	62 (45–85)	70 (50–89)	57 (45–79)	0.236
Initial mTICI grade				1.000
0	119 (98.3%)	39 (100.0%)	80 (97.6%)	
1	2 (1.7%)	0 (0.0%)	2 (2.4%)	
Successful recanalization	114 (94.2%)	35 (89.7%)	79 (96.3%)	0.210
Number of passes	2.0 (1.0–3.0)	2.0 (1.0–2.5)	2.0 (1.0–3.0)	0.906
Collateral grades				0.369
0–2	58 (47.9%)	21 (53.8%)	37 (45.1%)	
3–4	63 (52.1%)	18 (46.2%)	45 (54.9%)	
Local anesthesia	80 (66.1%)	24 (61.5%)	56 (68.3%)	0.463
EVT strategy				0.528
Stentriever + aspiration	89 (73.6%)	31 (79.5%)	58 (70.7%)	
ADAPT	23 (19.0%)	5 (12.8%)	18 (22.0%)	
Other	9 (7.4%)	3 (7.7%)	6 (7.3%)	
Rescue treatment	45 (37.2%)	17 (43.6%)	28 (34.1%)	0.315
Balloon angioplasty	23 (19.0%)	5 (12.8%)	18 (22.0%)	0.232
Stenting	37 (30.6%)	11 (28.2%)	26 (31.7%)	0.696
Artery occlusion site				0.525
ICA	62 (51.2%)	21 (53.8%)	41 (50.0%)	
M1	53 (43.8%)	15 (38.5%)	38 (46.3%)	
M2/ACA	6 (5.0%)	3 (7.7%)	3 (3.7%)	
Stroke subtype				0.067
LAA	59 (48.8%)	16 (41.0%)	43 (52.4%)	
CE	36 (29.8%)	17 (43.6%)	19 (23.2%)	
Other	26 (21.5%)	6 (15.4%)	20 (24.4%)	

NPAR, neutrophil percentage-to-albumin ratio; TIA, transient ischemic attack; mRS, modified Rankin Scale; ASPECTS, Alberta Stroke Program Early CT Score; SBP, systolic blood pressure; NIHSS, National Institutes of Health Stroke Scale; PLT, platelet count; AST, aspartate aminotransferase; ALT, alanine aminotransferase; OTT, time from symptom onset to thrombolysis; OPT, time from symptom onset to puncture; ORT, time from symptom onset to recanalization; PRT, time from puncture to recanalization; mTICI, modified thrombolysis in cerebral infarction score; EVT, endovascular therapy; ADAPT, A Direct Aspiration First Pass Technique; ICA, internal carotid artery; M1, main trunk of the middle cerebral artery; M2, first-order branch of the main trunk of the middle cerebral artery; ACA, anterior cerebral artery; IQR, interquartile range; LAA, large artery atherosclerosis; CE, cardioembolism.

**Figure 1:**
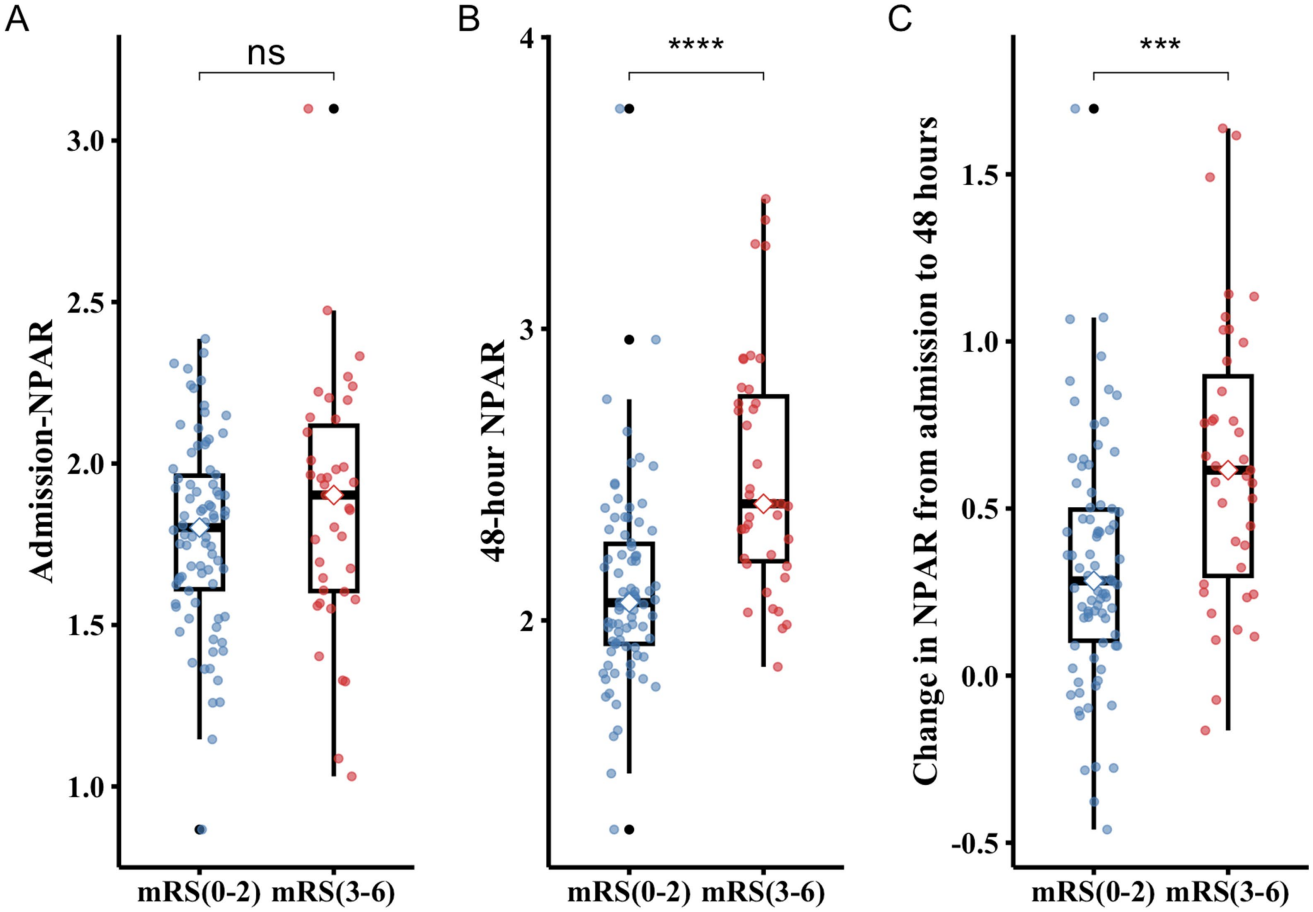
Box plots show admission **(A)**, 48-hour NPAR level **(B)**, and change in NPAR **(C)** in groups. The boxes represent the interquartile range (IQR), with the horizontal line indicating the median. Whiskers extend to 1.5 times the IQR, and individual points represent outliers. Statistical significance: *p < 0.05; **p < 0.01; ***p < 0.001; ****p < 0.0001; and ns, not significant.

Figure 1 shows box plots of admission NPAR, 48-h NPAR, and change in NPAR stratified by functional outcome groups. Levels of 48-h NPAR and changes in NPAR from admission to 48 h were significantly higher in the poor functional outcome group (mRS 3–6) than in the good functional outcome group (mRS 0–2) (all *p* < 0.001).

### 48-h NPAR levels were more correlated with the 3-month clinical outcome

**Figure 2:**
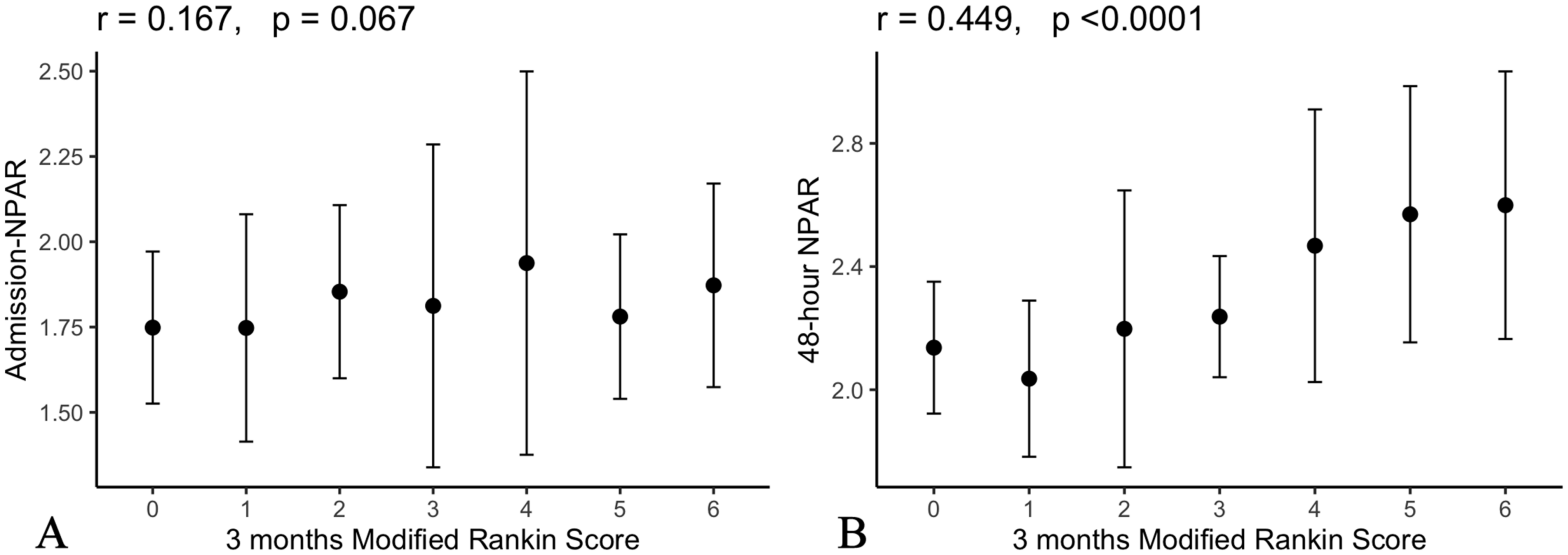
Correlation between admission **(A)** and 48-hour **(B)** NPAR levels and 3-month clinical outcome.

Higher 48-h NPAR levels were associated with poorer functional outcomes on univariate analysis, as reflected by the 3-month mRS score (*r* = 0.45, *p* < 0.0001; Figure 2A).

No significant correlation was observed between admission NPAR and the 3-month mRS score (*r* = 0.16, *p* = 0.067; Figure 2B).

### Univariable and multivariable logistic regression analyses

As shown in Table 2, univariable analyses indicated that baseline ASPECTS, NIHSS score, serum AST level, stroke subtype, symptomatic intracranial hemorrhage, 48-h NPAR, and changes in NPAR from admission to 48 h were significantly associated with poor functional outcome at 90 days (*p* < 0.05).

**Table 2:** Univariate logistic regression analysis.

Variable	Crude model for poor outcome at 90 days	
	OR (95%CI)	*p*-value
Demographic characteristics		
Age	1.04 (1.00–1.08)	0.059
Male	0.44 (0.15–1.13)	0.104
Vascular risk factors		
Current smoker	1.25 (0.55–2.96)	0.601
Current drinker	0.94 (0.31–3.23)	0.922
Hypertension	1.24 (0.58–2.72)	0.583
Diabetes	1.40 (0.57–3.32)	0.452
Atrial fibrillation	1.75 (0.65–4.58)	0.255
Prior stroke or TIA	1.42 (0.64–3.16)	0.385
Clinical assessment		
Pre-mRS	1.39 (0.46–4.00)	0.533
ASPECTS	0.76 (0.61–0.94)	**0.014**
Baseline SBP	1.00 (0.99–1.02)	0.545
Baseline NIHSS	1.18 (1.09–1.32)	**<0.001**
Intravenous thrombolysis	0.49 (0.15–1.34)	0.188
OTT	1.00 (1.00–1.01)	0.297
OPT	1.00 (1.00–1.00)	0.310
ORT	1.00 (1.00–1.00)	0.415
PRT	1.01 (0.99–1.02)	0.234
Admission laboratory data		
Blood glucose (mg/dL)	1.01 (1.00–1.01)	0.055
Neutrophil percentage	1.01 (0.98–1.04)	0.576
Albumin (g/L)	0.94 (0.85–1.04)	0.210
Platelet	1.00 (0.99–1.00)	0.290
Hemoglobin (g/L)	1.01 (0.98–1.03)	0.627
Fibrinogen (g/L)	1.21 (0.81–1.81)	0.352
ALT (U/L)	1.01 (0.99–1.04)	0.387
AST (U/L)	1.05 (1.02–1.09)	**0.007**
Total protein (g/L)	1.00 (0.93–1.07)	0.955
Globulin (g/L)	1.05 (0.96–1.15)	0.278
Creatinine (μmol/L)	1.01 (0.99–1.02)	0.439
NPAR levels		
Admission	2.16 (0.67–7.42)	0.204
At 48 h	19.27 (5.63–82.88)	**<0.001**
Change from admission	8.25 (2.85–27.65)	**<0.001**
Variables associated EVT		
Initial mTICI grade	NA	0.988
Successful recanalization	0.33 (0.06–1.58)	0.163
Number of passes	1.13 (0.85–1.52)	0.387
Collateral grades	0.70 (0.33–1.51)	0.370
Anesthesia	0.74 (0.34–1.66)	0.464
Intra-arterial thrombolysis	1.18 (0.52–2.81)	0.696
Rescue treatment		
Balloon angioplasty	1.49 (0.68–3.26)	0.316
Stenting	1.91 (0.69–6.19)	0.237
Artery occlusion site		
ICA	Reference	
M1	0.77 (0.34–1.70)	0.521
M2/ACA	1.95 (0.34–11.36)	0.436
EVT strategy		
Stentriever + aspiration	Reference	
ADAPT	0.52 (0.16–1.45)	0.236
Other	0.94 (0.19–3.81)	0.928
Stroke subtype		
LAA	Reference	
CE	2.40 (1.01–5.82)	**0.048**
Other	0.81 (0.26–2.29)	0.695
sICH	10.00 (2.82–47.19)	**<0.001**

NPAR, neutrophil percentage-to-albumin ratio; TIA, transient ischemic attack; mRS, modified Rankin Scale; ASPECTS, Alberta Stroke Program Early CT Score; SBP, systolic blood pressure; NIHSS, National Institutes of Health Stroke Scale; PLT, platelet count; AST, aspartate aminotransferase; ALT, alanine aminotransferase; OTT, time from symptom onset to thrombolysis; OPT, time from symptom onset to puncture; ORT, time from symptom onset to recanalization; PRT, time from puncture to recanalization; mTICI, modified thrombolysis in cerebral infarction score; EVT, endovascular therapy; ADAPT, A Direct Aspiration First Pass Technique; ICA, internal carotid artery; M1, main trunk of the middle cerebral artery; M2, first-order branch of the main trunk of the middle cerebral artery; ACA, anterior cerebral artery; CI, confidence interval; IQR, interquartile range; LAA, large artery atherosclerosis; CE, cardioembolism; sICH, symptomatic intracranial haemorrhage.Bold values indicate statistical significance.

Table 3 shows the results of the multivariable logistic regression models for poor functional outcome at 90 days. After controlling for baseline ASPECTS, NIHSS score, stroke subtype, baseline serum AST level and symptomatic intracranial hemorrhage, higher 48-h NPAR level (Model 1: aOR = 15.09, 95% CI 3.72–61.22, *p* < 0.001), and increase in NPAR from admission to 48 h (Model 2: aOR = 7.69, 95% CI 2.06–28.70, *p* = 0.002) were independently associated with poor functional outcomes. In both multivariable models, lower ASPECTS, higher baseline NIHSS scores, elevated AST levels, and the occurrence of sICH were all independently associated with an increased risk of poor functional outcome at 90 days. No significant association was observed between admission NPAR levels and 3-month clinical outcomes.

**Table 3:** Multinomial logistic regression models for poor functional outcome at 90 days.

Variable^a^	Model 1^b^		Model 2^c^	
	aOR (95%CI)	*p*	aOR (95%CI)	*p*
Baseline ASPECT	0.74 (0.55–0.99)	**0.045**	0.75 (0.57–0.98)	**0.037**
Baseline NIHSS	1.15 (1.02–1.29)	**0.026**	1.18 (1.04–1.33)	**0.008**
Baseline AST	1.05 (1.01–1.10)	**0.025**	1.05 (1.01–1.10)	**0.024**
Stroke subtype				
LAA	Reference		Reference	
CE	0.97 (0.28–3.44)	0.966	0.93 (0.27–3.18)	0.911
Other	0.26 (0.06–1.16)	0.076	0.38 (0.10–1.49)	0.166
sICH	7.62 (1.43–40.53)	**0.017**	6.55 (1.39–30.89)	**0.018**
NPAR levels				
At 48 h	15.09 (3.72–61.22)	**<0.001**	NA	
Change from admission	NA		7.69 (2.06–28.70)	**0.002**

NPAR, neutrophil percentage-to-albumin ratio; CI, confidence interval; ASPECTS, Alberta Stroke Program Early CT Score; NIHSS, National Institutes of Health Stroke Scale; AST, aspartate aminotransferase; sICH, symptomatic intracranial hemorrhage.

a Only variables with a *p*-value of <0.05 in univariable logistic regression are listed.

b Model 1 was adjusted for baseline ASPECT, baseline NIHSS, AST, stroke subtype, sICH, and 48-h NPAR.

c Model 2 was adjusted for baseline ASPECT, baseline NIHSS, AST, stroke subtype, sICH, and change in NPAR from admission to 48 h.Bold values indicate statistical significance.

### Cutoff value of NPAR for predicting poor functional outcomes

**Figure 3:**
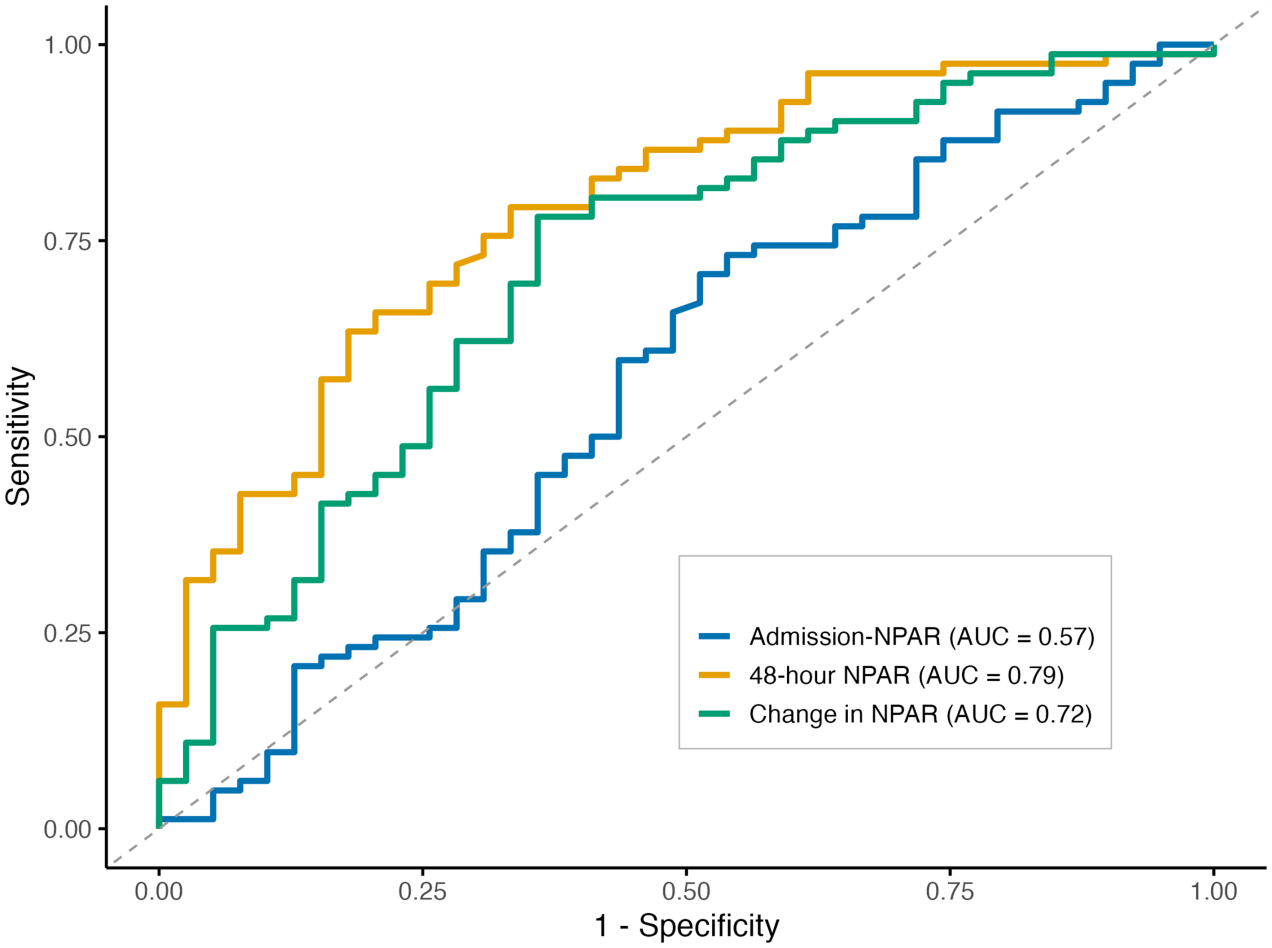
Receiver operating characteristic curves for neutrophil percentage-to-albumin ratio (NPAR) in predicting poor functional outcome at 90 days.

Figure 3 shows the receiver operating characteristic curves assessing the predictive performance of NPAR levels at admission, at 48 h, and the change in NPAR from admission to 48 h for poor functional outcome. The 48-h NPAR level demonstrated significantly superior discriminative ability for poor functional outcome (AUC = 0.79, 95% CI 0.70–0.88) compared with admission NPAR (AUC = 0.57, 95% CI 0.45–0.68; *p* < 0.001). By contrast, although the change in NPAR from admission to 48 h exhibited moderate discriminative performance (AUC = 0.72, 95% CI 0.62–0.82), its discriminative ability did not differ significantly from that of admission NPAR (*p* = 0.116). The optimal cutoff value of 48-h NPAR for discriminating poor functional outcomes was 2.312, with a sensitivity of 66% and a specificity of 79% (Supplementary Table S1).

## Discussion

Our study shows that a higher NPAR measured 48 h after EVT, as well as its dynamic change, were independent predictors of 3-month poor functional outcomes after EVT for acute anterior circulation large vessel occlusion stroke.

The neutrophil-to-albumin ratio has been associated with cardiovascular disease, sepsis, and intracerebral hemorrhage (17–19). However, these components play distinct roles in inflammation and disease processes. Higher neutrophil levels have been associated with poor prognosis (20–22), whereas higher albumin levels may exert protective effects and are therefore associated with favorable clinical outcomes (8, 23). While separate analyses may fail to capture their opposing effects, a combined assessment may better reflect their interaction and its association with different clinical conditions. NPAR, as an integrated biomarker, has been associated with clinical outcomes in AIS. Two recent retrospective studies found that elevated NPAR at admission was significantly correlated with poor 90-day functional outcomes in patients undergoing EVT (24, 25). Furthermore, Lu et al. (9) reported that higher NPAR was independently associated with increased all-cause mortality at 30 days, 90 days, 1 year, and during hospitalization.

Unlike previous studies, our data have shown no significant association between admission NPAR and 3-month clinical outcomes. One potential explanation is that admission NPAR primarily reflects baseline inflammatory and nutritional status, which may be influenced by comorbidities and variability in time from symptom onset, thereby limiting its predictive value. Within 6–8 h of stroke onset, neutrophils rapidly accumulate around cerebral vessels and initiate tissue infiltration (26). Following endovascular recanalization, neutrophil accumulation in ischemic and reperfused regions occurs at an accelerated rate and has been shown to correlate with poor neurological outcomes and greater brain injury severity (27). Hypoalbuminemia is common in patients with AIS and has been independently associated with poor functional outcomes, particularly in those undergoing EVT (28). Therefore, dynamic assessment of NPAR may provide stronger prognostic value than single measurements, and data on dynamic NPAR changes in patients undergoing EVT remain limited.

Our findings regarding the associations of 48-h NPAR and its dynamic changes with poor functional outcomes in patients with LVO-AIS may be explained by several mechanisms. One potential explanation is that 48-h NPAR may act as an alternative inflammatory biomarker reflecting peak inflammatory responses after EVT. Previous studies have shown that, during the first 48 h after stroke onset, impaired distal perfusion may promote endothelial inflammation, resulting in tissue injury and early blood–brain barrier disruption (15), while neutrophil infiltration typically peaks approximately at days 1–3 (16). In the context of EVT, prior studies have reported that vascular recanalization is accompanied by accelerated neutrophil recruitment in ischemic regions, which is associated with unfavorable outcomes (27). Substantial evidence demonstrates that proinflammatory responses are closely associated with poor clinical outcomes, particularly in patients treated with EVT (29–31). In addition, neutrophils are recognized as an inflammatory biomarker associated with stroke outcomes (32), whereas albumin exhibits anti-inflammatory properties with neuroprotective effects (33). Another potential explanation is that a high 48-h NPAR level may contribute to the no-reflow phenomenon, thereby increasing the likelihood of futile recanalization. El Amki et al. (20) demonstrated that, even after successful thrombolytic recanalization, cortical perfusion may fail to be restored due to neutrophil-mediated microvascular obstruction. Furthermore, excessive neutrophil accumulation promotes the release of proinflammatory mediators that disrupt endothelial integrity and increase blood–brain barrier permeability, leading to post-ischemic edema (34), while albumin exerts neuroprotective effects by suppressing inflammatory responses and oxidative stress (35), inhibiting platelet aggregation (36), and reducing cytokine-mediated adhesion within the postcapillary microcirculation (37).

Our present study has several limitations. First, despite the prospective design of the DETECT2-CHINA registry, the present study is retrospective and observational, which may introduce confounding bias. Although multivariable analyses were performed to adjust for measured confounders, residual confounding from unmeasured or unknown factors cannot be entirely excluded; for example, infarct volume, a well-established predictor of clinical outcome, was not adjusted for in the analyses. Moreover, as the data were obtained from a single comprehensive stroke center participating in the DETECT2-CHINA registry, the findings may be subject to selection bias and limited external validity. Second, as this was an exploratory analysis with a limited number of outcome events, the multivariable models may be subject to overfitting and imprecision, and the estimated effect sizes should be interpreted with caution, despite sensitivity analyses demonstrating directionally consistent associations. Third, the strong associations of 48-h NPAR and its dynamic changes with poor functional outcome may reflect temporal alterations in post-ischemic inflammation described in prior studies; however, this interpretation remains speculative in the absence of direct physiological or imaging correlations. Finally, NPAR data collection was obtained only at admission and 48 h post-procedure. Serial measurements at multiple time points after EVT could provide a more comprehensive understanding of the prognostic value of NPAR in this population.

## Conclusion

In conclusion, this study demonstrated that elevated 48-h NPAR levels and dynamic increases in NPAR from admission to 48-h were associated with poor functional outcomes in patients with LVO-AIS undergoing EVT. These findings need to be validated in future studies.

## Data Availability

Data are available upon reasonable request to the corresponding author.
