# Supplementary material for: Association of the neutrophil percentage-to-albumin ratio after endovascular treatment and 3-month clinical outcomes
Source: Front Neurol. 2026 Feb 12;17:1768949. doi: 10.3389/fneur.2026.1768949 (PMC12935595; doi:10.3389/fneur.2026.1768949)
Supplement: Supplementary file 1 [file Supplementary_file_1.docx]

**SUPPLEMENTAL MATERIAL**

“Association of neutrophil percentage to albumin ratio after endovascular treatment with 3-month clinical outcome”

Figure S1. Study flowchart of patient enrollment.

LVO, large vessel occlusion; AIS, acute ischemic stroke; NPAR, neutrophil percentage-to-albumin ratio.


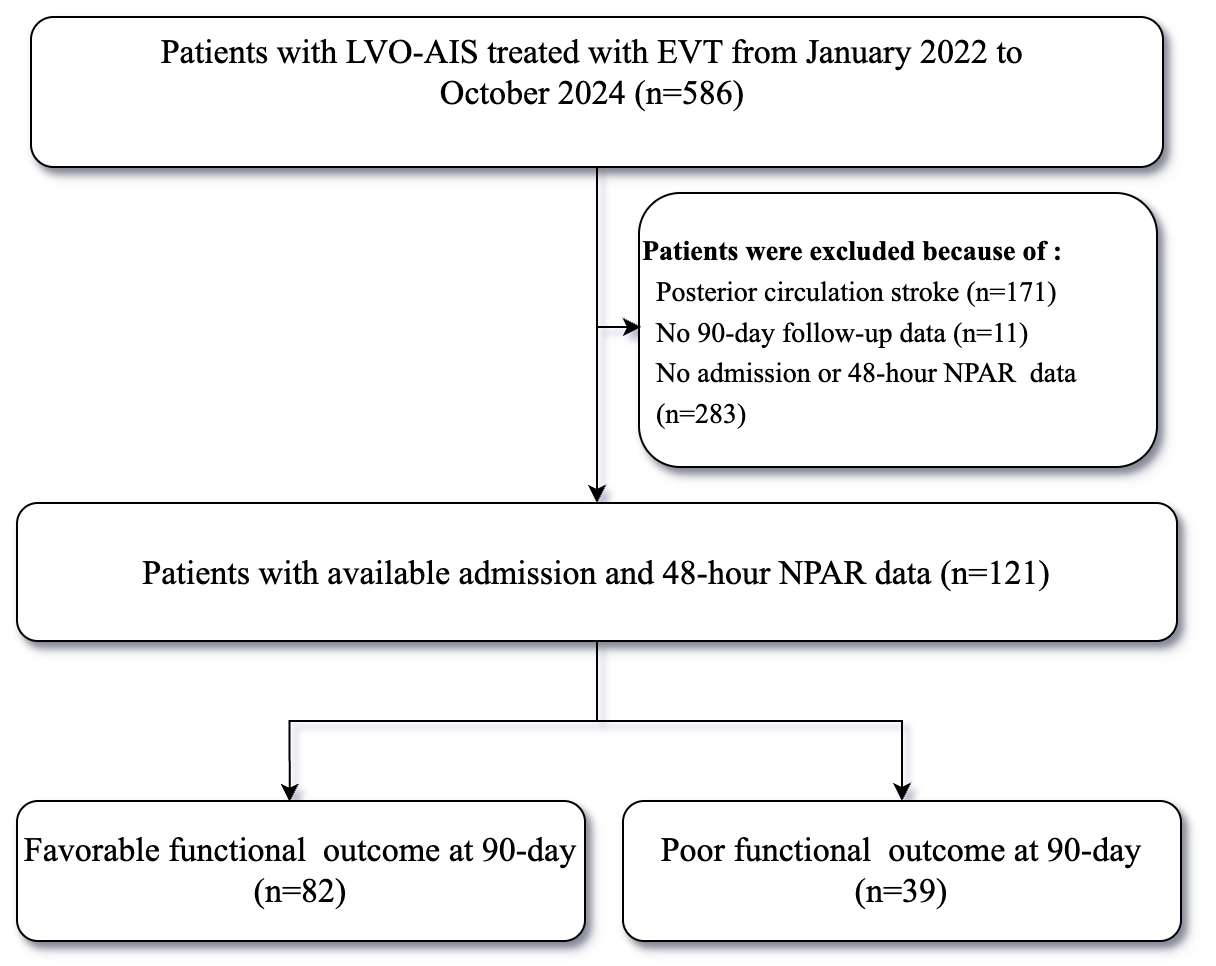


Table S1. Diagnostic performance of NPAR levels for predicting poor functional outcome at 90 days

| NPAR levels | AUC (95%CI) | Cutoff value | Sensitivity | Specificity | P value |
| --- | --- | --- | --- | --- | --- |
| At dmission | 0.57 (0.45-0.68) | 1.929 | 0.48 | 0.70 | Ref |
| At 48 hours | 0.79 (0.70-0.88) | 2.312 | 0.66 | 0.79 | **<.001** |
| Change from admission | 0.72 (0.62-0.82) | 0.513 | 0.64 | 0.78 | 0.116 |

Abbreviations: AUC, area under the receiver operating characteristic curve; CI, confidence interval.

Table S2. Sensitivity analysis for primary outcome.

| Variable | Model 1^a^ | | Model 2^b^ | |
| --- | --- | --- | --- | --- |
|  | OR (95%CI) | P | OR (95%CI) | P |
| Baseline ASPECT | 0.86 (0.65 to 1.13) | 0.273 | 0.78 (0.60 to 1.02) | 0.071 |
| Baseline NIHSS | 1.15 (1.03 to 1.29) | **0.014** | 1.17 (1.05 to 1.31) | **0.005** |
| sICH | 7.98 (1.80 to 35.38) | **0.006** | 6.80 (1.55 to 29.80) | **0.011** |
| NPAR levels |  |  |  |  |
| At 48 hours | 12.43 (3.35 to 46.11) | **<.001** | *NA* |  |
| Change from admisson | *NA* |  | 8.66 (2.45 to 30.60) | **<.001** |

Abbreviations: NPAR, neutrophil percentage-to-albumin ratio; CI, confidence interval; ASPECTS, Alberta Stroke Program Early CT Score; NIHSS, National Institutes of Health Stroke Scale; sICH, symptomatic intracranial haemorrhage.

^a^ Model 1 was adjusted for Baseline ASPECT, baseline NIHSS, sICH and 48-hour NPAR.

^b^ Model 2 was adjusted for Baseline ASPECT, baseline NIHSS, sICH and Change in NPAR from admission to 48 hours.

Table S3. Test of collinearity.

| Variable | Variance Inflation Factor | |
| --- | --- | --- |
|  | Model 1 | Model 2 |
| Baseline ASPECT | 1.117 | 1.140 |
| Baseline NIHSS | 1.055 | 1.062 |
| Baseline AST | 1.107 | 1.103 |
| Stroke subtype | 1.110 | 1.088 |
| SICH | 1.027 | 1.013 |
| NPAR levels |  |  |
| At 48 hours | 1.096 | *NA* |
| Change from admisson to 48 hours | *NA* | 1.081 |
